# Local perceptions of the impact of group interpersonal psychotherapy in rural Uganda

**DOI:** 10.1017/gmh.2016.15

**Published:** 2016-07-27

**Authors:** R. E. Lewandowski, P. A. Bolton, A. Feighery, J. Bass, C. Hamba, E. Haroz, V. Stavrou, L. Ndogoni, A. Jean-Pierre, H. Verdeli

**Affiliations:** 1 Department of Child and Adolescent Psychiatry, New York University, 1 Park Avenue, 7th Floor Reception, New York, NY, USA; 2 Department of International Health, Bloomberg School of Public Health, Johns Hopkins University, 615 North Wolfe Street, Baltimore, MD, USA; 3 Department of Health and Behavior Studies, Teachers College, Columbia University, 525 West 120th Street, New York, NY, USA; 4 Department of Mental Health, Bloomberg School of Public Health, Johns Hopkins University, 615 North Wolfe Street, Baltimore, MD, USA; 5 World Vision Uganda, Plot 15B, Nakasero Road, P.O. Box 5319 Kampala, Uganda; 6 Columbia Group for Children in Adversity, 5 River Road, Suite 116 Wilton, CT, USA; 7 World Vision East Africa Regional Office, P.O. Box 133 – 00502 Karen, Nairobi, Kenya; 8 Department of Counseling and Clinical Psychology, Teachers College, Columbia University, 525 West 120th Street, New York, NY, USA

**Keywords:** Community, community impact, depression, education, group interpersonal psychotherapy, low and middle-income countries (LMICs), rapid ethnographic assessment, Uganda

## Abstract

**Background.:**

This study investigated local perceptions of changes stemming from a long-standing Group Interpersonal Psychotherapy (IPT-G) program for the treatment of depression in rural Uganda. The study was conducted in a low-income, severely HIV/AIDS-affected area where in 2001 the prevalence of depression was estimated at 21% among adults.

**Method.:**

Data were collected using free-listing and key informant qualitative interviews. A convenience sample of 60 free-list respondents was selected from among IPT-G participants, their families, and other community members from 10 Ugandan villages. Twenty-two key informants and six IPT-G facilitators were also interviewed.

**Results.:**

Content analysis yielded five primary categories of change in the community related to the IPT-G program: (1) improved school attendance for children; (2) improved productivity; (3) improved sanitation in communities; (4) greater cohesion among community members; and (5) reduced conflict in families. Community members and IPT-G facilitators suggested that as depression remitted, IPT-G participants became more hopeful, motivated and productive.

**Conclusion.:**

Results suggest that providing treatment for depression in communities with high depression prevalence rates may lead to positive changes in a range of non-mental health outcomes.

Greater attention and resources have recently been devoted to improving mental health care in low and middle income countries (LMIC), though this support has not matched efforts in other areas of health and development (Miranda & Patel, 2005; Prince *et al*. 2007; Saxena *et al*. 2007; Eaton *et al*. 2011). Mental health, however, is an essential component in efforts to achieve major health and development goals (Miranda & Patel, 2005; Prince *et al*. 2007; WHO, 2010). Depression, for example, is a leading cause of disability (WHO, 2001; Murray *et al*. 2012), associated with negative economic and health outcomes including reduced productivity and role functioning (Spitzer *et al*. 1995; Judd *et al*. 2000; Bolton *et al*. 2002, 2004; Lund *et al*. 2010), poor prognosis and treatment adherence in HIV/AIDS (Ickovics *et al*. 2001; Cook *et al*. 2004). Children of mothers with depression have higher risk of mental disorders, are more likely to be low birth-weight, under-nourished, and are less likely to be vaccinated or attend well-child visits (Patel *et al*. 2003; Rahman *et al*. 2004; Weissman *et al*. 2006; Hanlon *et al*. 2009).

The association between depression and these negative outcomes suggests that increasing availability and access to depression treatments may have broad health and developmental benefits. There remains, however, little evidence that depression treatment translates into such improvements (WHO, 2005; Lancet Global Mental Health Group, 2007; Lund *et al*. 2011). Therefore, the current study explored perceptions of local people in rural Uganda about changes that took place in their community, beyond the reduction of symptoms and improved functioning, as a result of a group interpersonal psychotherapy (IPT-G) depression treatment program.

## IPT-G in Uganda

In 2002, researchers from Johns Hopkins University and Columbia University were invited to the rural Ugandan districts of Masaka and Rakai by the non-governmental humanitarian organization World Vision International to adapt and evaluate the group format of Interpersonal Psychotherapy (IPT-G), a manualized treatment for depression (Weissman *et al*. 2000). World Vision speculated that unidentified factors related to the HIV/AIDS epidemic, possibly having to do with mental health, were impeding the uptake and success of their development programming (Bolton *et al*. 2009). A qualitative assessment identified two depression-like illnesses, *Yo'Kewkyawa* (self-hatred) and *Okwekubagiza* (self-pity), that were seen by local people as important problems related to HIV/AIDS (Wilk & Bolton, 2002; Bolton *et al*. 2003).

Based on these findings, Bolton and colleagues adapted and tested IPT-G, delivered by trained lay people from the local communities (Bolton *et al*. 2003; Verdeli *et al*. 2003). Compared with wait-listed controls, people who received IPT-G showed greater reduction in depression symptoms and were less likely to be depressed following treatment. These intervention effects were maintained at a 6-month follow up (Bass *et al*. 2006). Between 2002 and 2004, the IPT-G facilitators who provided treatment in this trial continued to lead IPT-G groups as volunteers under the supervision of World Vision. In 2004, these facilitators were hired by World Vision and IPT-G became the centerpiece of the Masaka-Rakai Psychosocial Project (MRPP), part of World Vision's formal psychosocial programming for the region.

In the MRPP, IPT-G was supplemented with an income generating activity (IGA), which included a 3-day entrepreneurial training and supervised preparation of a business plan and financial grant application. The IGA was provided several weeks after the end of the 16-week IPT-G treatment. In 2007, due to high local demand for IPT-G, World Vision trained 40 local volunteers to deliver IPT-G treatment under regular supervision from World Vision staff facilitators. At this time, an HIV education component was added to the initial phase of the treatment and all IGA activities were discarded, except for the entrepreneurial training, which took place approximately 1 month after treatment for those who desired it.

As before, people were invited to join the IPT-G treatment program only if they met the locally validated criteria for depression, as assessed by screening and a standard diagnostic interview. At the completion of the 16-week IPT-G treatment, many groups continued to meet as peer-led support groups, some for years. At the time of this study (10–31 May 2010), the MRPP consisted only of the IPT-G treatment and the entrepreneurial training, the HIV education component having been discontinued. The MRPP concluded in July 2010 with the end of World Vision's 15-year funding cycle for operations in the region. From the introduction of IPT-G in Masaka and Rakai in 2002 to the end of the MRPP, it was estimated that over 5900 individuals received IPT-G treatment for depression (Ndogoni, personal communication, 27 July 2012). In this study, former IPT-G participants, their family members, and members of the community at large were asked in qualitative interviews to report from their perspective what changes resulted from the IPT-G program for them and their community.

## Method

This study employed a rapid qualitative research method that included free-listing and key informant interviews (Wilk & Bolton, 2002). A qualitative method was selected so that local respondents could report any changes they perceived. Interviews were conducted in Luganda, the local language, by 10 local university-educated Ugandan interviewers who could speak and write English. Interviewers completed 3 days of training in qualitative interviewing techniques provided by the first author (REL), which focused on accurate recording of participants’ responses and the use of non-leading questions and probes. Interviewers wrote down responses verbatim. Interviewers read the consent form, written in Luganda, out loud to all respondents and obtained verbal consent for their participation. Respondents were assured during the consent process that neither their decision whether or not to participate, nor the answers responses they provided, would affect their access to support from World Vision and other organizations. Interviewers did not record participants’ names or other identifying information. No one approached to be a respondent in this study refused to participate.

A convenience sample of 10 villages representing different geographical locations and population sizes was chosen from among those in which the MRPP was currently active. There was no reliable record of how long IPT-G groups had been run in each of these communities. Due to logistical challenges and resource limitations it was not possible to return to any of the villages where the 2002 IPT-G evaluation had been conducted. The institutional review board at Teachers College, Columbia University approved this study.

### Free-listing interviews

Free-listing interviews were used to identify a range of changes resulting from the IPT-G groups as perceived by the local population. A convenience sample of 60 free-list respondents was selected (six per village), representing both sexes and a range of adult ages. Respondents were individuals who had participated in the IPT-G program, members of their households (spouses or other relatives, including young adults who were children at the time when a caregiver participated in the IPT-G program), and members of the community at large (see Table 1 for a distribution of the Free List respondents). Each respondent was asked four questions: (1) What has changed for people who took part in the IPT-G groups?; (2) What has changed for families of people who took part in the IPT-G groups?; (3) What has changed for children in families of people who took part in the IPT-G groups?; (4) What has changed in the whole community because of the IPT-G groups? Respondents were asked to list all of the changes they could think of. Interviewers asked for a brief description of each response and probed exhaustively in an open-ended way for additional changes.

**Table 1.** Distribution of respondents in free-list interviews

Respondent type	Male	Female	Total
IPT-G participant	16	10	26
Family member	5	17	22
Adult child	6	0	6
Community member	7	0	7
(Age range: 18–64)			*n* = 60

### Key informant interviews

To gather greater depth of information, the five categories of changes most frequently reported in free-listing interviews were explored further in key informant interviews, in the following format: ‘Last week, people in this community said _(insert reported change based on results of free-listing) because of the IPT-G groups. Can you tell me more about this change?’ Key informants were 22 community members (13 men; 9 women; 2 or 3 per village) ranging in age from 28 to 63, who were identified by free-list respondents as knowledgeable about specific changes reported. Interviewers used open-ended, non-leading probes to encourage respondents to talk in depth about areas of interest.

### IPT-G facilitator interviews

Interviews were conducted with a convenience sample of six current volunteer IPT-G facilitators (3 female; 3 male) representing 15% of the total number of facilitators practicing at the time, selected to represent the range of geographical locations within the program area. Interviews with IPT-G facilitators aimed to triangulate responses given by IPT-G participants, family members, and community members, and to learn more about their perceptions of the mechanisms underlying the changes reported. Facilitators were asked the following question, which was developed during the fieldwork in response to information gathered during free-list interviewing: ‘Community members who participated in this study mentioned things such as cash rounds, trainings, and improvements in productivity as being related to the IPT-G groups. Can you tell me about this?’ Cash rounds are a common method of raising money in which group members contribute a small sum to a pot that is then given to a different member each week to pay for large expenses like school fees or funeral arrangements. They are not a prescribed component of IPT-G but are a common practice in development programs, church meetings, and other informal gatherings, which predated the arrival of IPT-G in the region. ‘Trainings,’ refers to other development related information and programming delivered by World Vision and other organizations.

The lead author conducted all facilitator interviews with assistance from an experienced translator. English translations of facilitator responses were transcribed verbatim by the interviewer.

### Data analysis

To achieve consensus, interviewers working in pairs collaboratively coded data from the interviews they conducted. They were supervised by two authors to ensure fidelity to coding procedures. Responses were summarized into lists that included every discrete response and the number of times that response was mentioned. All interviewers together and two authors then reviewed these lists to obtain consensus for responses that expressed the same concept even though the wording was different. Conceptually equivalent items were combined into a single item and the number of responses summated. Interviewers and authors then sorted the responses into categories of changes, which became the focus of key informant interviews. Key informant interviews and facilitator interviews were analyzed in the same way, summing the frequency with which individual and conceptually equivalent responses were given.

## Results

Free-list respondents reported five general categories of changes that happened because of the IPT-G program: (1) school attendance for children improved; (2) productivity increased in agriculture and animal husbandry; (3) sanitation in communities improved; (4) togetherness and unity improved; and (5) conflict in families was reduced. Responses grouped in each category are described in sections below. Descriptions are based on data from both free-listing and key informant interviews. Responses were included in the following sections if they were given by at least 20% of free-list respondents (*N* = 12 minimum) or key informants (*N* = 5 minimum).

### School attendance for children improved

The most frequent response given in free-list interviews was that because of the IPT-G treatment program, children go to school more because people now pay school fees (34 of 60 respondents; Table 2). Eleven of 22 key informants reported that children are now encouraged to work hard with their parents to increase family income in order to afford school fees, and 10 explained that parents now prioritize saving money to pay school fees. Six key informants stated that group members help each other to provide school fees and supplies so that children can go to school. Five key informants also stated that group members advised each other about various means of acquiring money to pay for school fees. Four key informants reported that IPT-G groups helped members ‘to put worries aside’ and to concentrate on paying school fees for their children.

**Table 2.** Summary of all changes reported in free-list interviewing, mentioned by more than one person. (Primary Free List Question: What are all the changes that have happened for people who participated in the IPT-G groups?)

Change in community (60 respondents)	Number of respondents who mentioned change
People pay school fees for children to go to school	34
People are active in agriculture and animal husbandry. Cleanliness in families has improved	33
	28
We get enough food from farming	26
People are working harder	21
We received knowledge and skills in modern farming, agriculture and animal husbandry	21
Cleanliness in the community has improved	20
Children now go to school due to changed attitudes and motivation in children	19
Parents learn to behave well and respect other family members	18
Behaviors in homes have improved	16
We get counseling and advice concerning our problems from fellow group members	16
People behave well, as society expects	14
There is peace in families	13
We still lack some support	13
We give each other advice about animal and crop husbandry and how to overcome problems	13
There is unity and cooperation	12

Nineteen of 60 free-list respondents mentioned that children now go to school because of changed attitudes of parents and increased motivation in children. The most frequent response of key informants, given by 12 of the 22, was that IPT-G groups ‘opened people's eyes’ and helped them to realize the value of sending their children to school (Table 3). These key informants elaborated that before participating in the IPT-G groups, parents ‘never minded about children because of their problems’ and that children did not go to school ‘because of *Okwekubagiza*’ (self-pity). One key informant, expressing a concept representative of what was reported by others, explained that a community member might have thought, ‘Why should we send children to school since we will soon be dead, and it doesn't matter?’ This key informant elaborated that group members came to recognize that even though they themselves may soon die, their children will continue to live and must be prepared for their future through education.

**Table 3.** Responses given by key-informants asked to give more information about the change reported by community members — ‘Children go to school’

Responses (22 respondents)	Number of respondents who mentioned change
IPT-G groups opened people's eyes and they realized the value of sending their children to school instead of leaving them at home to provide labor: (before groups parents never minded about children, because of their problems, and in the groups they were taught the importance of education; before group teachings they were very lazy and reluctant and did not know what to do and in these groups they talked about the issues that stopped them from working so they were able to get solutions and resumed to strongly devote themselves to work; before children did not go to school because of *okwekubagiza*; we thought, why should we send children to school, because we will be dead and it does not matter)	12
We encourage children to work hard with their parents in order to increase the family income (to pay for school fees)	11
We try as much as we can to save money and use it sparingly in order to pay fees for our children	10
We support each other with availing children with school requirements and school fees to enable them go to school	6
We speak with children in friendship and have convinced them to return to school	5
We advised each other about various means of acquiring money to pay fees for our children	5
Children can now go to school because they have improved health (with the arrival of IPT-G, when a child is sick, people do not stay sleeping, they now see the importance of seeking medical assistance)	4
IPT-G has enabled us to inspire parents to take their children to school (to instill the spirit and zeal in those who did not have it)	4
People participate in school meetings and to know how their children are performing in schools	4
We put our worries aside and concentrated on paying fees for our children	4
We removed conflicts from families through encouraging parents to discuss their differences. This enabled children to return to school.	3
We encourage parents who are not able to afford expensive schools to take their children to Universal Primary Education schools (government schools) so they can study	2
We advise group members to reduce drinking alcohol in order for children to go to school	2
People now have enough food for their children to eat and they take food at schools for their children to eat instead of cash	2
We get examples from our fellow members who have educated their children	2
We advised ourselves not to marry many women because it is an obstacle to children's education	1
We follow up children who are not going to school and punish them with local leaders and the police and they go back to school	1
We look for better schools for our children to study from	1
We organized football competitions in schools	1
We discussed with teachers about children's problems	1
The leader of the IPT-G group gave children scholastic materials	1
We give gifts to children who have performed well	1
We discovered that some people were not to be blamed for their children not going to school	1

Other key informants reported that parents now ensure that their children do go to school. Five key informants stated that parents now ‘speak with children in friendship,’ which has enabled them to persuade their children to attend school again. Four key informants explained that IPT-G has enabled group members to inspire parents to take their children to school and, as one key informant stated, to ‘instill the spirit and zeal’ in those children who did not have it. Four key informants explained that parents now participate in meetings at school to ensure their children are doing well. Finally, four key informants indicated that children miss school less often due to illness because their parents no longer ‘stay sleeping’ and seek medical assistance when needed. A dramatic example of IPT-G members’ renewed commitment to education was a group of women who had lost their spouses and children to AIDS. At the completion of their 16-week IPT-G treatment this group began to care for orphans and child-headed households in their community. The women also founded a school for children in the community, and monitored local families to ensure that children attended.

### Productivity increased in agriculture and animal husbandry

Four of the six most frequently reported free-listing responses were related to increased productivity (Table 2). Free-list respondents stated that because of the IPT-G groups: participants are active in agriculture and animal husbandry (33 of 60 respondents); families now produce sufficient food through farming (26 respondents); participants are working harder (21 respondents); and members have received knowledge in modern farming, agriculture and animal husbandry (21 respondents). Other changes related to productivity reported by free-list respondents included: ‘We give each other advice about animals and crop husbandry and how to overcome problems’ (13 respondents).

Key informant interviews suggested that increased productivity is attributable to three factors stemming from the IPT-G groups: (1) improved knowledge and skills in modern farming methods; (2) increased cooperation and social support around farming; (3) increased emotional strength and hopefulness. The most frequent response, given by 19 of 22 key informants, was that group members learned improved agriculture practices. The four next most frequent key informant responses highlight the sharing of farming information between group members that stems from ‘togetherness among people.’

The sixth most frequent key informant response (6 respondents), which was the most frequent response that did not refer directly to farming practices, was that group participants had been ‘restored’ by the counseling in the groups and were able to work well. These key informants explained, for example, that before IPT-G groups, many community members were ‘lazy and reluctant’ and ‘not able to do anything’ because they were ‘thinking too much and worrying about problems.’ They elaborated that previously members believed it served nothing to work hard because their land was too small to be productive.

### Sanitation in communities improved

The third most frequent free-list response was ‘Cleanliness in families has improved (28 of 60 respondents). Similarly, 20 free-list respondents reported, ‘Cleanliness in the community has improved because there are better sanitation facilities and practices’ (Table 2). Key informants reported that sanitation improved because people are building toilets and kitchens and constructing racks for dishes and utensils (17 of 22 key informants), clearing rubbish from the family compound (12 respondents) and practicing better hygiene, such as hand washing and boiling drinking water (11 respondents).

Key informants reported that IPT-G group members now visit each other and discuss cleanliness and monitor sanitation and hygiene (8 respondents). Five key informants reported that group members make an effort to assist each other in communal work to improve sanitation, for example by helping to build toilets.

### Togetherness and unity improved

Free-list respondents reported that because of the IPT-G groups people have learned to give and receive counseling and advice about their problems (16 respondents; Table 2). Respondents stated that there is greater unity and cooperation because of the IPT-G groups (12 respondents). Almost all key informants reported that group members learned to support each other in their problems, and care about each other as before they had not (20 respondents; Table 4). Eight key informants reported that members now advise each other on how to overcome problems (8 respondents). Key informants reported that community members now ‘work together’ and do not segregate along lines of wealth, religion, and tribe (11 respondents), and that they have been taught to work together as a group to grow crops and rear animals (9 respondents). Key informants further reported that they have been able to resolve conflict, remove jealousy, and see each other as equals (7 respondents). Seven key informants stated, ‘Hatred has stopped.’ Finally, key informants reported that ‘visiting and knowing each other’ has increased (7 respondents), and that members now trust each other because they vowed not to spread problems to outsiders (5 respondents).

**Table 4.** Responses given by key-informants asked to give more information about the change reported by community members

Responses (22 respondents)	Number of respondents who mentioned change
In IPT-G groups members have learned to help and support each other in problems (we have learned to care about each other, we love each other like brothers, before we did not care and with the group now we care)	20
People work together without segregating each other along lines of rich and poor, religion, tribe	11
We were taught to work together as a group in growing groups and rearing animals	9
We advised each other on how to overcome problems	8
IPT-G has helped to resolve conflicts and removed jealousy because we see each other as equals	7
Visiting and knowing each other in the area has increased	7
There was advice and emphasis that love in the families is very important and necessary	5
We have vowed not to spread our problems to outsiders so we now trust each other	5
We learned that all people are the same (equal): each person has an equal voice, rich or poor	3
IPT-G has developed in us interest in good things we see from our friends	2
We give each other financial support in the group	2
We were taught to forgive each other in the group as well as in the families	2
We implement whatever we have agreed upon to do	2
We get courage from those who have managed to overcame their problems	2
The groups taught us to use non-verbal communication like giving gifts	1
We stopped being suspicious of each other and we got peace	1
We counsel our group members who are in problems	1
We stopped to worry and now we are happy	1
We learned to make good crafts like mats and baskets	1
Those who went through IPT-G started to advising the rest of the community	1
They taught us to live or to put aside thinking so much about dying of aids and leaving our young children	1
They discouraged us from divorcing so as not to leave our children and families	1
They are taught to praise those who have done good things	1
We help each other to acquiring seeds and animal feed	1
We are concerned that our children are well	1

### Conflict in families was reduced

Free-list respondents reported that ‘parents learned to behave well and respect other family members’ (18 of 60 respondents; Table 2) and that ‘behaviors in homes’ have improved (16 respondents). Thirteen free-list respondents reported that there is ‘peace in families.’ Key informants reported that members now advise each other in groups on how to resolve family conflicts (12 of 22 respondents; Table 5) and are encouraged to maintain peace and love in their families and to respect all family members (6 respondents). Additionally, key informants reported that group members learned to work hard and were too busy for family arguments (10 respondents). Seven key informants stated that group members advised themselves to change and ‘leave bad behaviors.’

**Table 5.** Responses given by key-informants asked to give more information about the change reported by community members: ‘Conflict has been reduced; peace in families has increased’

Responses (22 respondents)	Number of respondents who mentioned change
We advise each other in the group on how we can resolve our respective family conflicts	12
We learnt to work hard in our families which kept us busy and had little time for arguments	10
We advised ourselves to change and leave bad behaviors	7
People are encouraged to maintain peace and love in their families and to respect the family members	6
IPT-G groups taught us to stop drinking alcohol so that we can avoid the problems that come along with drunkenness	4
Working together and looking for money together as husband and wife has increased development and unity in families	4
We advise and reconcile those who have wrong each other	4
We encourage our group members to maintain trust in their families	4
We work together in our families to get what we need at home	3
We learned to separate important things from those that are not important. We are now not easily offended	3
We learnt to keep our family secrets	3
Women now know their husbands’ jobs and their incomes	3
Counseling brought back hope in people and they were restored to their normal senses	3
We stopped quarreling in public	3
We removed bad thoughts from our minds	2
We visit ourselves in our homes and we get to know about our family problems and advise each other accordingly	2
People are afraid to die and leave their children as orphans	2
We learnt to talk about issues and to compromise with our husbands	2
We were taught to be patient and forebear our spouses when they are in the wrong	2
Good communication between husband and wife reduces conflicts in families	2
we talk to men about their responsibilities in families	2
Them are less fights and quarrels in families because people have now some money	2
We taught our children to behave well	2
we were advised to avoid envying each other and others	2
We learned to take care of our husbands, to welcome them back and to prepare for them good meals (our duties)	2
We were taught to do for our husbands things that make them happy	2
We refer people to other community members who are more knowledgeable than us	1
People infected with HIV/AIDS are advised to go to hospitals for help	1
There are less diseases (sickness) in families and this has reduced conflicts in family members	1
Togetherness with husband and wife is good because each is free to share his/her thoughts which minimizes misunderstandings	1
We instill into ourselves the heart of not mistreating our wives, but to treat them well	1
People reduced their levels of anger, which was a major cause of fighting in families	1
We were taught that every person is useful to another	1
People were taught to love each other and to forgive each other	1
We advise ourselves in our groups about our problems without blaming (judging) each other	1
We realized our own personal faults and mistakes	1
Wives were allowed to by their husbands to receive their personal visitors	1
We were taught not to be greedy	1
We were taught to avoid listening to rumors or gossiping	1
We are taught to be creative in our families	1
We learned to give our husbands respect	1
We have learned to find the right time to deliver criticism to our spouses	1

## IPT-G facilitator interviews

### Facilitator-reported changes due to participation in IPT-G treatment

All six IPT-G facilitators reported that IPT-G group members ‘were happy.’ Five of six facilitators reported that ‘now members work.’ In many cases, facilitators’ responses suggest that being ‘happy’ was directly related to being able to resume working. Three facilitators reported that ‘members’ problems have been resolved by the group suggestions.’ Three facilitators also stated that formerly depressed group members resumed socializing. Two facilitators stated that husbands and wives now ‘work together’ and that this was associated with increased productivity and increased happiness. Facilitators also referred to material gains stemming from IPT-G participation. For example, two facilitators reported that members were able to ‘buy land and livestock’ and that members now ‘have food’ and ‘food to feed their children’ as a result of farming. Two facilitators reported that members have resumed their businesses, for example selling pancakes or baskets. Responses that members ‘have money,’ and ‘built a house,’ or have ‘improved production’ were given by one facilitator each.

### Interrelation of IPT-G, development-related trainings, and cash rounds

Three of six facilitators stated that development-related information, for example, about modern farming techniques, sanitation, and the idea to start cash rounds, were introduced by group members themselves near the end of treatment. Facilitators stated that members drew on their own expertise and shared information among themselves about raising animals. Three facilitators emphasized that focusing on weekly and long-term goals in the treatment led to these other ideas, and two explained that the focus on long-term non-mental health related goals, like planting seeds to grow fruit and vegetables, began in the middle phase of treatment. Two facilitators indicated that at times they made suggestions based on their experience leading other trainings, including in farming methods. Two other facilitators reported that their groups focused only on treating depression, while another two stated that other ideas and practical goals were introduced but that this occurred as group members’ depression began to remit, and they started ‘to look for what to do.’ In her description, one facilitator explained: ‘Before treating depression, treating the mind, they couldn't do the others and they were even fearing. It was after going through treatment for the mind, after opening up…and getting suggestions and starting working, they started to get other ideas.’ Another added, ‘When people were still depressed, (development trainings) would come and people would say ‘this cannot help me’ and they ignored it. Before they didn't want to have it because they were depressed.’

## Discussion

The IPT-G depression treatment program was reported by respondents to have led to a range of changes in general quality of life and local development. Two of these changes in particular, increased school attendance and increased productivity in farming, align with major international development priorities, articulated by the United Nations in the Millennium Development Goals, to achieve universal primary education, and eradicate extreme poverty and hunger (United Nations, 2013). Free-list and key informant interviews suggested two interrelated mechanisms underlying the reported changes: (i) increased social support and strengthened social bonds, (ii) the treatment and remission of depression. Also, the communication of farming and sanitation information appeared to be an important contributor to reported improvements in productivity and cleanliness.

The impact of increased support and social ties among IPT-G participants and between participants and other family and community members, which were themselves a main category of change, was interwoven in the other categories of change as well. For the category ‘school attendance has improved,’ key informants stated that group members inspired each other to take children to school and supported each other in helping children to have the necessities for school. For the category ‘productivity has increased,’ responses indicated that togetherness brought new farming practices and that people visited each other, learned new farming techniques, and helped with ‘digging.’ For the category ‘sanitation has improved,’ changes were linked to increased cooperation and sense of responsibility for others.

Similarly, apparent reductions in depression symptoms were highlighted in explanations of multiple categories of reported changes. For example, key informants reported that IPT-G group members who ‘never minded about children because of their problems,’ learned to put ‘worries aside and concentrate on paying school fees.’ Agricultural productivity improved because people who previously could ‘not do anything’ and ‘had no interest in digging’ because of ‘thinking too much’ and ‘worrying about their problems,’ were ‘counseled and…restored’ and could ‘work well’ at farming. A number of other free list and key informant respondents gave answers consistent with this mechanism, though not with enough frequency to meet the cut-off for inclusion in the results. For example, five free list respondents reported having ‘more strength’ to work and being ‘motivated and encouraged.’ Three key informants reported that counseling eased family conflict by renewing hope and restoring people to their ‘normal senses.’ Two others reported that sanitation and hygiene improved because ‘people stopped thinking a lot,’ and no longer ‘felt sorry for themselves.’ Free list respondents also mentioned mental health specific outcomes related to the IPT-G program, such as ‘There is no more depression,’ and ‘Now we are happy,’ which also were not reported frequently enough to include in the results. Finally, three or fewer free list respondents reported negative changes due to the IPT-G program, including husbands or wives of participants fearing their partners will find other men or women as a result of treatment, and jealousy among community members who did not participate in the IPT-G groups.

During the lifespan of the IPT-G program in Masaka and Rakai, World Vision and other organizations led concurrent initiatives to improve education, farming, and sanitation that may have contributed to the reported changes, though it was not possible to determine precisely which other programs had been received by specific respondents or communities. The description of apparent mechanisms underlying the reported changes does however support the specific contribution of the IPT-G program. World Vision staff also reported anecdotally that numerous development programs operated in the area long before IPT-G was introduced but were not successful, explaining that it was the added influence of the IPT-G program that helped participants benefit from other programs. IPT-G facilitator responses about the interplay between IPT-G and other development programs clearly reflected this concept. IPT-G facilitator responses also suggested that information from other programs did not alter the IPT-G group focus on the treatment of depression.

Though the current study did not systematically explore factors that contributed to the longevity and local acceptability of the IPT-G program, these features have important implications for the sustainability of mental health programming globally. The authors speculate that critical elements included the initial emphasis on establishing meaningful and sustained local partnerships and engaging the local population to define important problems. The IPT-G program was selected and tailored to respond to specific needs of the local population, which were identified through extensive formative research and refined in close partnership with community leaders and the local aid organization. Major multinational initiatives that are currently disseminating manualized mental health care packages can maximize ecological validity and community engagement by investing thoroughly in these foundational steps; if these steps are deemphasized for the sake of expediency, programs may risk reduced impact and sustainability.

### Limitations

Results of this study must be interpreted cautiously for several reasons, in particular, the challenge of isolating IPT-G program effects from those of other programs operating in the area. Additionally, the entrepreneurial training offered as part of the MRPP may have contributed to reported changes, although most reported changes were not entrepreneurial in nature. Whenever possible interviewers selected free-list respondents that had completed the 16-week IPT-G treatment but had not yet participated in the entrepreneurial training, so that their responses could be more confidently attributed to the IPT-G component of the program.

The sampling approach may also have introduced a positive response bias. For logistical reasons, IPT-G facilitators helped to identify respondents. Those respondents who were former IPT-G participants may have reported more positive changes out of a sense of loyalty or obligation to the facilitator. Respondents were assured during the consent process that the research team equally valued positive and negative responses, and that respondents’ access to support from World Vision and other organizations would not be affected by the answers they gave. According to World Vision staff, respondents in this study were aware of the impending termination of the IPT-G treatment program in Masaka and Rakai and may have emphasized positive changes to persuade World Vision to continue the IPT-G program.

The sampling approach also limits the generalizability of the results. Most respondents were IPT-G participants and their family members, thus it is uncertain when responses referred to participants and their families or to the community as a whole. In small communities, where many community members participated in IPT-G treatment, or had a family member participate, the changes reported by participants and family members may in fact be representative of changes perceived in the community as a whole. In larger communities, not all community members may recognize the changes reported by participants and their families.

Finally, IPT-G facilitator responses highlighted questions about IPT fidelity. While volunteer facilitators appeared to be guided by the IPT treatment model, whereby therapy focuses on problem solving in one of four areas (grief, role transitions, role disputes, interpersonal deficits), the extent to which they faithfully employed IPT treatment techniques was less clear. Facilitators emphasized general group therapy facilitation techniques, which are also practiced in IPT-G, including drawing on group members for suggestions for each others’ problems and refraining from ‘giving answers’ to the members. There was likely variability among volunteer facilitators in fidelity to IPT techniques; nevertheless, all facilitators reported that IPT-G retained a primary focus on depression treatment, rather than on farming, or other topics related to material gain. The effectiveness of IPT in treating depression is likely greatest if adherence to the model and techniques is explicitly retained. It is also plausible, however, that the evolution of group content in Uganda to match group members’ priorities played an important part in its longevity and sustained importance. The results of this study suggest that as task-shifting approaches increasingly become the foundation of strategies to improve mental health treatment in low and middle-income countries, programs should emphasize supervision to ensure ongoing fidelity to evidence-based principles, while allowing the flexibility to optimize the local validity of the intervention over time. It is also practically essential and ethically imperative to consider and respect the financial and time constraints of the non-specialist providers that will play a central role in sustaining mental health programs over the long term.

## Conclusion

This study indicated that the IPT-G depression treatment program was perceived by respondents to have led to positive changes in quality of life and development for those who received treatment, their family members, and their communities. Two of the reported changes are consistent with Millennium Development Goals to achieve universal primary education and eradicate extreme poverty and hunger, suggesting that treating depression in low and middle-income countries may have a positive impact on development efforts. Because multiple programs operated simultaneously in the IPT-G program area, it may be most appropriate to consider the reported changes as stemming from the combined impact of the IPT-G treatment and other programs: a picture of what might be expected to result over time from a sustained, coordinated aid effort that includes a treatment response for depression-like illness. As links between mental health and development become more widely accepted, and development donors become increasingly interested to support mental health programming, evaluations of mental health initiatives should continue to assess impact on priority development outcomes and processes, especially in ways that can be captured with quantitative data.
